# Hidden reach of the micromanagers

**DOI:** 10.1186/1741-7007-8-53

**Published:** 2010-05-11

**Authors:** Peter S Linsley, Aimee L Jackson

**Affiliations:** 1 Regulus Therapeutics, 1896 Rutherford Road, Carlsbad, CA 92008, USA

## Abstract

Small interfering RNAs can trigger unintended, microRNA-like off-target effects, but the impact of these effects on functional studies has been controversial. A recent study in *BMC Genomics *shows that microRNA-like effects can predominate among the 'hits' of functional genomics screens.

See research article http://www.biomedcentral.com/1471-2164/11/175

## Commentary

The development of RNA interference (RNAi) techniques ranks as one of the major technical advances of recent years in experimental biology. These techniques have provided investigators with powerful tools for disrupting gene expression with unprecedented ease. At long last, functional genetic screens in cultured cells are possible. While these screens are now widely used, they often yield 'off-target hits', where reproducible functional readouts occur in the absence of disruption of the intended target gene. A recent report by Sudbery *et al. *[1] in *BMC Genomics *describes a systematic investigation of off-target hits in an RNAi screen for modulators of apoptosis induced by TNF-related apoptosis inducing ligand (TRAIL-induced apoptosis). The results unexpectedly show that off-target hits are enriched for common sequence motifs resembling the targeting sites ('seed regions') of microRNAs (miRNAs), endogenous noncoding RNAs that repress expression of mRNAs. These findings suggest technical improvements in screening methodologies using small interfering RNAs (siRNAs), and on a broader level, demonstrate the powerful ability of miRNAs to modulate biological pathways.

## microRNA-like off-target silencing by siRNAs

One of the more popular embodiments of RNAi technology involves the use of siRNAs, which are readily used for gene silencing in a variety of cultured cells [2]. The impact of their discovery is evidenced by the fact that an entire industry has developed to supply standardized siRNAs and the reagents for their use. While siRNAs are usually designed to silence perfectly matched specific mRNA targets, they can also silence multiple unintended targets. Silencing of unintended targets occurs when siRNAs act like miRNAs, which repress expression of specific mRNAs by binding short target sequences in their 3' untranslated regions (UTRs) that match the miRNA 'seed regions' (nucleotides 1 to 8). Because of the short lengths of these targeting sites, they are found frequently in transcripts and an individual miRNA can therefore have many targets. Unintended, miRNA-like silencing by siRNAs also involves target sites that match siRNA seed regions, and also involves many transcripts. While perfectly matched targets often are silenced robustly (up to around tenfold), miRNA-like off-target regulations are weak (generally less that twofold). Because of their low magnitude, miRNA-like off-target regulations are most easily detected using statistical techniques that measure small changes in the expression of many genes. miRNAs modulate expression of most mammalian genes and have been termed 'micromanagers of gene expression' [3].

miRNA-like off-target silencing has been well documented [4], but its consequences in siRNA functional screens are not well understood. Even applying technical standards for calling hits in siRNA screens [5], it is still common to find off-target screen hits. It was noted that top screen hits may result from off-target effects mediated by shared sequence identity in siRNA seed regions [6]. However, a systematic global examination of the effects of miRNA-like off-target silencing in siRNA screens has not been reported.

## Enrichment of seed-sequence motifs in siRNA screening hits

Sudbery *et al. *[1] performed an siRNA screen for modulators of TRAIL-induced apoptosis in HeLa cells. The screen utilized a sub-genomic siRNA library designed to target the 'druggable genome' (that is, that part of the genome encoding potential drug targets such as protein kinases, G-protein coupled receptors, and so on), and one caveat to these studies is that the results might be biased in some way by the subset of sequences selected for the library. While the technical quality of the screen was good, and Sudbery *et al. *found that siRNAs to several well-documented apoptosis pathway members behaved as expected in the assay, they also found that a large fraction of the top-scoring siRNAs was confirmed as off-target hits.

These findings are reminiscent of previous findings by Lin *et al. *[6] that screening enriches for siRNAs with specific seed sequences. When Sudbery *et al. *examined their top-scoring siRNAs, they found repeated occurrences of several seed sequences, including seeds found in the human miRNAs miR-26a, miR-145 and miR-384. Of particular importance, the addition of a miR-26a seed hexamer motif, ACTTGA, to an inert sequence specifically conferred protection against TRAIL-induced apoptosis. In addition, *bona fide *miR-26a, miR-145 and miR-384 sequences blocked TRAIL-induced apoptosis in a number of cell types. Taken together, these findings suggest that these screening experiments enriched for siRNAs with miRNA-like off-target activity. In other words, a high-scoring siRNA hit was more likely to possess miRNA-like activity than activity against a single gene target.

## miRNA-like versus single-target hits

The results of Sudbery *et al. *[1] suggest ways to improve siRNA screens. It is common practice to call hits from screens only after at least two different siRNAs that target the same gene yield the same result [5]. However, Sudbery *et al*. show that different siRNAs can sometimes trigger the same miRNA-like off-target phenotype. While such examples would be expected to be relatively uncommon, they do indeed occur. This suggests that a better criterion for hit calling is to verify the phenotype with two independent siRNAs not present in the original siRNA library.

Another area for potential improvement is in siRNA library design. The propensity for miRNAs to trigger observable phenotypes suggests that sequences matching miRNA seed sequences should be filtered out during library design. Filtering must be balanced against the need to maintain sufficient sequence diversity space for selecting sequences with optimal on-target activity. These considerations suggest that more work will need to be done to optimize siRNA library design parameters. The results from this study should provide a strong incentive for investigators to purchase libraries from vendors who use design techniques to avoid miRNA-like off-target effects.

Technical concerns aside, one of the striking findings from the study of Sudbery *et al. *[1] was how strongly and frequently miRNA-like hits scored in their screen. Eight off-target hits (three of which match a seed hexamer of miR-26a) gave stronger phenotypes than the top on-target hit, MYC-associated factor X (MAX). Moreover, transfer of the miR-26a seed hexamer motif to a negative control siRNA conferred a phenotype as strong as that of the positive control used in the assays, an siRNA targeting caspase 8. Thus, seed pairing may be sufficient for target recognition by miRNAs, in agreement with the most common type of conserved miRNA target site in mammals [7]. The top-scoring hits in the screen described by Lin *et al. *were also miRNA-like [6].

miRNA-like off-target hits can, therefore, often score more favorably in siRNA screens than siRNAs that target single genes. Why should this be? Perhaps siRNA design algorithms remain suboptimal, so that many siRNAs do not optimally silence their intended targets. Another possibility is that triggering a given phenotype is a function of the number of tests being made. Because miRNA-like activity of siRNAs involves the regulation of so many genes, perhaps there is a better chance of scoring simply because there are more 'shots on goal'.

**Figure 1:**
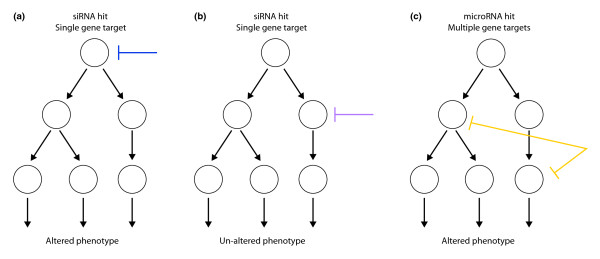
**A model for the propensity for off-target hits in siRNA screens**. Shown is a hypothetical, directed acyclic network or pathway with multiple branches contributing to a complex phenotype. This scheme resembles current views on extrinsic and intrinsic apoptotic pathways that drive cell-survival decisions [10]. **(a)** Some siRNAs may be effective at disrupting a pathway. An siRNA targeting a root node (blue line) will disrupt all downstream flow through the pathway. **(b)** Other siRNAs may be ineffective at disrupting a pathway. For example, an siRNA targeting a daughter node (magenta) will be ineffective at inhibiting downstream flow because it does not block all parallel paths to the phenotype. **(c)** microRNA-like hits (yellow) may be more effective pathway blockers. Because microRNAs have multiple targets, they can block multiple paths to a phenotype.

Another possibility to explain selection for miRNA-like hits is that the miRNA mode of target regulation is more effective at modulating complex phenotypes (Figure 1). siRNAs targeting single genes may effectively block flow through a pathway at certain points, but not at others. Unaffected target nodes in a network could, for example, represent points where there are parallel paths to a phenotype. On the other hand, miRNA-like hits may be more effective because they are able to block multiple paths toward the measured phenotype(s). miRNA-like regulation is likely to recapitulate natural and evolutionarily selected modes of pathway control.

Selection of miRNA hits may therefore reflect the role of miRNAs as natural micromanagers of biological pathways [3]. A corollary of this idea is that an siRNA that does not match a true miRNA would be likely to trigger a spectrum of off-target regulations that have not been selected during evolution. Importantly, miRNA seed matches are disproportionally represented relative to non-miRNA seed matches in the hit lists from Sudbery *et al. *[1]. miRNA seed hexamers comprise around 25% (4 of 16) of significantly scoring seed hexamers, but only around 8% (348 of 4,096) of all possible hexamer words (hypergeometric *P*-value approximately 9e-3). This is consistent with the notion that certain miRNAs have been selected during evolution to regulate TRAIL-induced apoptosis.

The studies of Sudbery *et al. *[1] are important because they open up new ways of thinking about siRNA screening results. They suggest ways to improve hit calling and siRNA library design. Moreover, they implicate siRNA screens as an experimental tool for examining the role of miRNAs on pathway regulation. It will be important to extend these studies to other siRNA functional screens, including full genome-scale screens [8]. With full genome screens, it may be possible to identify key miRNA targets by matching single gene screen hits with targets of miRNA sequences overrepresented in off-target hits. This approach has been used to identify multiple miRNA targets whose silencing by siRNAs triggers a cell-growth phenotype [9]. The study of Sudbery *et al. *suggests that further surprises lie ahead as to the extent that miRNA micromanagers affect biological pathways.
